# Pending challenges to e-mental health in the COVID-19 era: Acceptability of a smartphone-based ecological momentary assessment application among patients with schizophrenia spectrum disorders

**DOI:** 10.1192/j.eurpsy.2021.920

**Published:** 2021-08-13

**Authors:** J.-D. Lopez-Morinigo, B.-E. Maria Luisa, A. Porras-Segovia, A. Sánchez-Escribano Martínez, P.-J. Escobedo-Aedo, V. González Ruiz-Ruano, L. Mata-Iturralde, L. Muñoz-Lorenzo, S. Sánchez-Alonso, A. Artés-Rodríguez, E. Baca-Garcia

**Affiliations:** 1 Psychiatry, Universidad Autónoma de Madrid, Madrid, Spain; 2 Psychiatry, Universidad Autonoma, Madrid, Spain; 3 Psychiatry, Hospital Universitario Fundación Jiménez Díaz, Madrid, Spain; 4 Signal Theory And Communications, Universidad Carlos III, Madrid, Spain

**Keywords:** Schizophrenia spectrum disorders, acceptability, ecological momentary assessment

## Abstract

**Introduction:**

Concerns have been raised about ecological momentary assessment (EMA) acceptability among patients with schizophrenia spectrum disorders (SSD), which is of major relevance during the e-Mental health-focused COVID-19 pandemic.

**Objectives:**

To investigate i) the levels of adherence to a passive smartphone-based EMA tool, the Evidence-Based Behavior (eB2), among SSD patients; and ii) putative predictors of this.

**Methods:**

Sample: SSD (F20-29-ICD10) outpatients, age 18-64, without financial incentives, recruited over 17/06/2019-11/03/2020 at the Hospital Universitario Fundación Jiménez Díaz (Madrid, Spain). Those who accepted the eB2 installation -users- and those who did not -non-users- were compared in sociodemographic, clinical, premorbid adjustment, neurocognitive, psychopathological, insight and metacognitive variables by a multivariable binary logistic regression model.

**Results:**

Sample (N=77): n=41 males; age: 47.69±9.76 years, n=24 users (31.2%). n=14 users (70%) had the eB2 installed at follow-up (median=14.50 weeks).

**Table:** 

Multivariable binary logistic regression model on ‘user’ as outcome						
	β	SE	Wald	p	OR	95% CI
Age	-0.075	0.038	3.910	0.048	0.928	0.861-0.999
Education level	-0.967	1.289	0.563	0.453	0.380	0.030-4.755
Early adolescence premorbid adjustment	-0.285	0.110	6.695	0.010	0.752	0.606-0.933
Trail Making Test A	-0.030	0.025	1.488	0.222	0.970	0.924-1.018
Trail Making Test B	-0.005	0.010	0.278	0.598	0.995	0.976-1.014
Cognitive Insight	0.062	0.061	1.043	0.307	1.064	0.944-1.200

X^2^=25.296,df=6,p<0.001. Nagelkerke-R^2^=44.7%. Correctly classified: 76.9%, users:54.5%, non-users:88.4%.

**Conclusions:**

Acceptability of a smartphone-based EMA application among SSD patients was low. Age (young) and good premorbid adjustment predicted acceptability. e-Mental Health methods need to be tailored for patients with SSD. Otherwise, these highly vulnerable individuals may be neglected by e-health-based services in the post-COVID-19 years ahead.

